# Dynamics of contrast-frequency characteristics of the visual system in patients with schizophrenia

**DOI:** 10.1192/j.eurpsy.2023.967

**Published:** 2023-07-19

**Authors:** M. A. Tumova, I. I. Shoshina, V. V. Stanovaya, Z. T. Guseynova, M. V. Ivanov

**Affiliations:** 1 Institute for Cognitive Research, St. Petersburg State University; 2 Biological therapy of the mentally ill, V.M. Bekhterev National medical research center psychiatry and neurology, Saint Petersburg, Russian Federation

## Abstract

**Introduction:**

Visual impairment in schizophrenia is of interest as a potential biomarker of the mental state. The study of visual impairment in patients with schizophrenia is difficult due to the fact that visual perception can be influenced not only by the patient’s condition, but also by age, drug treatment, concomitant eye diseases, etc. To reduce the influence of these factors, we studied visual disturbances in schizophrenic patients in dynamics at the second and eighth weeks of stable antipsychotic single treatment.

**Objectives:**

To reveal changes in visual impairment in inpatients with schizophrenia on the background of changes in mental state.

**Methods:**

Eleven inpatients with schizophrenia who received antipsychotic monotherapy and thirteen healthy subjects of the same age and sex were included in the study. Examinations were performed at weeks 2 and 8 of treatment. The contrast-frequency characteristics of the visual system were examined using computer visocontrastometry. Visocontrastometry was performed in Gabor element contrast detection (gratings) with spatial frequencies of 0.4, 0.6, 0.8, 1.0, 4.0, 10.0 and 17.9 cycles/degree. Images of Gabor elements of different spatial frequency were repeated in random order 8 times each. The severity of the mental state was assessed during the interview using the PANSS (Positive and Negative Syndrome Scale).

**Results:**

Patients’ total PANSS score at week 2 averaged 94.09±17.58 and at week 8 averaged 52.45±6.06; at week 8 the total score was significantly lower than at week 2 (V = 66, p-value = 0.004). In the low-frequency region after treatment, patients tended to have lower thresholds (V = 2207, p-value = 0.060), but both at week 2 and week 8, thresholds were significantly higher in patients than in the healthy group (W = 7233, p-value < 2.2e-16, W = 6924.5, p-value = 1.204e-11, respectively). Mid-range frequencies increased at week 8 compared with week 2 (V = 925, p-value = 0.003), but were also lower at weeks 2 and 8 than in the healthy group (W = 1479, p-value = 7.247e-12, W = 3156.5, p-value = 0.004, respectively). In the high frequency region, thresholds also increased after the treatment (V = 908, p-value = 2.084e-05), at week 2, thresholds in patients were significantly lower than in healthy controls (W = 2574.5, p-value = 2.757e-07), and at week 8, thresholds in the patient and healthy groups were not different (W = 4759.5, p-value = 0.461).

**Conclusions:**

The impairments in the low spatial frequencies in schizophrenic patients appear earliest and, apparently, are the most persistent. Changes in the middle and high frequencies appeared to be more variable with changes in the mental state. Unfortunately, our design does not allow us to judge the persistence of the revealed changes. Further prospective studies are needed to investigate the relationship of visual disturbances with other symptoms.

**Disclosure of Interest:**

None Declared

This work was supported by a grant from the Russian Science Foundation (project no. 22-18-00074).

